# Ras induces experimental lung metastasis through up-regulation of RbAp46 to suppress RECK promoter activity

**DOI:** 10.1186/s12885-015-1155-7

**Published:** 2015-03-25

**Authors:** Hsuan-Heng Yeh, Yu-Fen Tseng, Yu-Chiao Hsu, Sheng-Hui Lan, Shan-Ying Wu, Giri Raghavaraju, Da-En Cheng, Ying-Ray Lee, Tsuey-Yu Chang, Nan-Haw Chow, Wen-Chun Hung, Hsiao-Sheng Liu

**Affiliations:** 1 Department of Microbiology and Immunology, National Cheng Kung University, Tainan, Taiwan; 2 Institute of Basic Medical Sciences, National Cheng Kung University, Tainan, Taiwan; 3 Department of Medical Research, Chiayi Christian Hospital, Chiayi, Taiwan; 4 Department of Parasitology, National Cheng Kung University, Tainan, Taiwan; 5 Department of Pathology, National Cheng Kung University, Tainan, Taiwan; 6 Center of Infectious Disease and Signaling Research, National Cheng Kung University, Tainan, Taiwan; 7 National Institute of Cancer Research, National Health Research Institutes, Tainan, Taiwan

**Keywords:** RbAp46, Ras, RECK, Metastasis, Tumorigenesis

## Abstract

**Background:**

Mutant Ras plays multiple functions in tumorigenesis including tumor formation and metastasis. Reversion-inducing cysteine-rich protein with Kazal motifs (RECK), a metastasis inhibitor gene, suppresses matrix metalloproteinase (MMP) activity in the metastatic cascade. Clarifying the relationship between Ras and RECK and understanding the underlying molecular mechanism may lead to the development of better treatment for Ras-related tumors.

**Methods:**

Suppression subtractive hybridization PCR (SSH PCR) was conducted to identify Ha-*ras*^val12^ up-regulated genes in bladder cancer cells. Stable cell lines of human breast cancer (MCF-7-*ras*) and mouse NIH3T3 fibroblasts (7–4) harboring the inducible Ha-*ras*^val12^ oncogene, which could be induced by isopropylthio-β-D-galactoside (IPTG), were used to clarify the relationship between Ras and the up-regulated genes. Chromatin immunoprecipitation (ChIP) assay, DNA affinity precipitation assay (DAPA) and RECK reporter gene assay were utilized to confirm the complex formation and binding with promoters.

**Results:**

Retinoblastoma binding protein-7 (RbAp46) was identified and confirmed as a Ha-*ras*^val12^ up-regulated gene. RbAp46 could bind with histone deacetylase (HDAC1) and Sp1, followed by binding to RECK promoter at the Sp1 site resulting in repression of RECK expression. High expression of Ras protein accompanied with high RbAp46 and low RECK expression were detected in 75% (3/4) of the clinical bladder cancer tumor tissues compared to the adjacent normal parts. Ras induced RbAp46 expression increases invasion of the bladder cancer T24 cells and MMP-9 activity was increased, which was confirmed by specific lentiviral shRNAs inhibitors against Ras and RbAp46. Similarly, knockdown of RbAp46 expression in the stable NIH3T3 cells “7-4” by shRNA decreased Ras-related lung metastasis using a xenograft nude mice model.

**Conclusions:**

We confirmed that RbAp46 is a Ha-*ras*^val12^ up-regulated gene and binds with HDAC1 and Sp1. Furthermore, RbAp46 binds to the RECK promoter at the Sp1 site via recruitment by Sp1. RECK is subsequently activated, leading to increased MMP9 activity, which may lead to increased metastasis *in vivo*. Our findings of Ras upregulation of RbAp46 may lead to revealing a novel mechanism of Ras-related tumor cell metastasis.

**Electronic supplementary material:**

The online version of this article (doi:10.1186/s12885-015-1155-7) contains supplementary material, which is available to authorized users.

## Background

H-*ras*, K-*ras*, and N-*ras* proteins are members of a large superfamily of low-molecular-weight GTP-binding proteins which control signaling pathways that are key regulators of numerous aspects of normal cell growth and malignant transformation [1]. About 30% of human tumors express Ras point mutations [2]. At least three major effectors of Ras are responsible for downstream signal transduction, including the Raf/mitogen-activated protein kinase (MAPK) pathway, the phosphatidylinositol 3-kinases (PI3Ks) pathway and the Ral guanine nucleotide exchange factors (RalGEFs) pathway [3]. Contributions of these pathways are mainly observed in tumor initiation, such as cell survival, proliferation and transformation. However, little is known about their involvement in Ras-induced cell invasion and metastasis. Moreover, the roles of mediators in Ras induction of invasion and metastasis are not fully understood [4]. Therefore, the precise effects of Ras-related factors and their functions in tumorigenesis warrant further investigation.

The reversion-inducing cysteine-rich protein with Kazal motifs (RECK) gene is a membrane-anchored glycoprotein that negatively regulates matrix metalloproteinases (MMPs) and inhibits tumor metastasis and angiogenesis [5,6]. The RECK gene was first isolated as a transformation suppressor gene to induce flat reversion in a v-Ki-*ras-*transformed NIH3T3 cell line [5]. Further studies showed that RECK is down-regulated in many human cancers including pancreatic, breast, non-small cell lung cancer and osteosarcoma [7] and in cells that ectopically express active oncogenes [8]. It is known that histone deacetylation and promoter methylation are the mechanisms by which cancer cells act to suppress RECK expression [9,10]. However, the underlying mechanism of decreased RECK expression in Ras-related tumorigenesis is still unclear. Chang HC *et al*. showed that silencing RECK by Ras oncogene is mediated by DNA methyltransferase 3b-induced promoter methylation [10]. Recently, Loayza-Puch F *et al*. reported that hypoxia and Ras-signaling pathways cooperatively down-regulate RECK through *miR-372/373* and *miR-21* [11]. Interestingly, RECK promoter activity suppressed by Ras through Sp1 protein binding at Sp1 binding motif has been reported [12]. Chang CH *et al*. further demonstrated that induction of the Ha-*ras*^val12^ oncogene increases the binding between the HDACs-Sp1 complex and the Sp1 site on the RECK promoter to suppress RECK expression [13]. Therefore, a better understanding of the underlying mechanism that enables Ras expression to induce RECK suppression may lead to the development of better treatments for Ras-related tumors.

The retinoblastoma binding protein 46 (RbAp46) was initially identified as a Rb binding protein, as shown by Rb affinity column studies [14]. RbAp46 is expressed in the nucleus as a core component of histone deacetylase (HDAC) complexes: mSIN3, which is involved in global transcriptional repression, and NuRD, a multi-subunit complex exhibiting chromosome remodeling activity [15]. RbAp46 is also known as histone acetyltransferase (HAT) type B subunit 2, formed when RbAp46 binds selectively to histones H2A and H4; it greatly stimulates HAT activity by recruiting HDAC enzymes to the target promoter [16]. These studies indicate that RbAp46 plays important roles in modification and remodeling of chromatin during cell growth and differentiation. In addition, studies also revealed that RbAp46 acts as a tumor suppressor which inhibits the malignant phenotype of adenovirus-transformed human embryonic kidney (HEK) 293 cells in tumor formation and that RbAp46 arrests the cell cycle at the G2/M phase [17]. Furthermore, overexpression of RbAp46 suppresses tumorigenicity of neoplastigenic breast epithelial cells [18]. Constitutive expression of RbAp46 induces the epithelial-mesenchymal transition (EMT) in mammary epithelial cells [19]. Moreover, RbAp46 overexpression correlates with migration of lung cancer cells and serves as a prognostic marker for distant metastasis of non-small cell lung cancer [20]. All together, these studies indicate that RbAp46 plays an important role in the tumorigenesis of various cancers. We have identified RbAp46 as a Ras up-regulated gene using the IPTG inducible system in a bladder cancer cell line and a NIH3T3 fibroblast variant. It is known that RbAp46 can form a complex with HDAC to regulate gene suppression. Ras expression induces HDAC1 to bind with Sp1 and then suppresses RECK promoter activation via the Sp1 site. Therefore we were interested in investigating the roles of RbAp46 in Ras-induced RECK suppression and Ras-related tumorigenesis.

In this study, we demonstrated that Ras up-regulates RbAp46 expression and then down-regulates the metastasis suppressor RECK through association of RbAp46 with HDAC1 and Sp1. The RbAp46 could bind at the Sp1 site via association with Sp1 protein and subsequently repress RECK promoter activation. Importantly, we also found RbAp46 increases Ras-induced MMP-9 activity and metastasis. Our findings also showed clinical evidence of a relationship among Ras, RbAp46 and RECK in bladder cancer.

## Methods

### Cell lines

T24 (human bladder cancer cells), MCF-7 (human breast cancer cells) and the 7–4 cells (mouse NIH3T3 fibroblast derive) were maintained in Dulbecco’s modified Eagle’s medium (DMEM; Invitrogen Life Technologies, GIBCO, Gaithersburg, MD, USA) supplemented with 10% fetal bovine serum (GIBCO) at 37°C in a 5% CO_2_ incubator. T24 and MCF-7 cells were obtained from American Type Culture Collection (ATCC; Rockville, MD). The MCF-7-*ras* cells derived from MCF-7 contain an inducible Ha-*ras*^*val12*^ oncogene [21]. The 7–4 cells derived from mouse fibroblast NIH 3T3 cells contain the same inducible Ha-*ras*^*val12*^ oncogene as that in MCF-7-*ras* cells [22].

### Plasmids

The mouse RECK promoter-luciferase plasmid pGL3-RECK and Sp1 mutant plasmid, originally isolated by Dr. Noda M. (Kyoto University, Japan) [12], were kindly provided by Dr. Hung WC [23]. (National Sun Yat-Sen University, Taiwan). The full-length human RbAp46 gene (1278 base pairs) was amplified by RT-PCR. The primers used were RbAp46 forward 5′-ATGGCGAGTAAAGAGATGTT-3′ and RbAp46 reverse 5′-TTAAGATCCTTGTCCCTCCA-3′. The *Not*I site was used to clone the RbAp46 cDNA fragment into the pcDNA3.1 vector (Invitrogen, Carlsbad, CA, USA) to create the plasmid pcDNA-RbAp46. Genomic DNA extracted from transitional cell papilloma RT4 cells and HPV E7 immortalized uroepithelial cells were used to amplify RbAp46 promoter (Additional file 1).

### Cell lysis and Western blot analysis

The cell lysates were prepared as previously described [24]. Briefly, cells were harvested in lysis buffer (50 mM Tris–HCl, pH 7.4, 100 mM NaCl, 2 mM EDTA, 1% NP-40, 10 mM sodium orthovanadate, 0.1% SDS, 100 mM phenylmethylsulfonyl fluoride, 2 mg/ml aprotinin, 2 mg/ml pepstatin A, and 10 mg/ml leupeptin). Protein concentration was determined using the Bradford assay (Bio-Rad, Richmond, CA, USA). Equal amounts of cellular protein were loaded onto 10-12% sodium dodecyl sulfate (SDS) polyacrylamide gels (SDS-PAGE). Proteins were then transferred onto nitrocellulose membranes and the blots were probed with the following antibodies: anti-Ras antibody (Oncogene Science, La Jolla, CA, USA), anti-RbAp46 antibody (Calbiochem, Gibbstown, NJ, USA), anti-RECK antibody (MBL, Nagoya, Japan), anti-Sp1 and HDAC antibodies (Santa Cruz Biotechnology Inc., Santa Cruz, CA, USA) and anti-β-actin antibody (Sigma, St. Louis, MO, USA). The bands were detected using enhanced chemiluminescence kit (ECL) according to the manufacturer’s instructions (PerkinElmer, Boston, MA, USA).

### RNA extraction and RT-PCR

Total RNA of the cells was extracted using Trizol reagent (Invitrogen, Carlsbad, CA, USA). First-strand cDNA was synthesized from 0.2 μg to 1 μg of total RNA with the oligo-dT primer and the Moloney murine leukemia virus (MMLV) reverse transcriptase (Promega, Madison, WI, USA). The conditions for PCR were conducted with the RbAp46 or RECK primers for 30 cycles of denaturation (94°C/1 min), annealing (60°C/1 min) and extension (72°C/1 min), followed by 1 cycle of final extension (72°C/10 min). The conditions for β-actin were 25 cycles for denaturation (94°C/30 sec), annealing (55°C/30 sec) and extension (72°C/1 min), followed by 1 cycle of final extension (72°C/10 min). The primers used for RT-PCR were as follows: RbAp46 forward 5′-ATGGCGAGTAAAGAGATGTT-3′; RbAp46 reverse 5′- TGGAGGGACAAGGA-3′; RECK forward 5′-CCTCAGTGAGCACAGTTCAGA-3′; RECK reverse 5′-GCAGCACACACACTGCTGTA-3′; β-actin forward 5′-CCTGTGGCAT CCACGAAACT-3′; β-actin reverse 5′-GAAGCATTTGCGGTGGACGAT- 3′. After the reaction, PCR products were separated on a 2% agarose gel and detected under UV light.

### Real-time PCR

Real-time PCR reaction was performed in a total volume of 20 μl containing 2 μl cDNA, 10 μM each of forward and reverse primers, and 10 μl SYBR® Green PCR Master Mix (Applied Biosystems, Foster City, CA, USA). The cDNA in this reaction solution was denatured for 10 min at 95°C followed by 40 cycles of PCR, which included denaturing at 95°C for 15 sec and annealing/elongation at 60°C for 60 sec in the wells. Real-time PCR was performed on the StepOne real-time PCR System (Applied Biosystems, Foster City, CA, USA). The primers used for real-time PCR were as follows: RbAp46 forward 5′-GTAGGCGAGTAAAGAGATGT-3′; RbAp46 reverse 5′-TTAAGATCCTTGTCCCTCCA-3′; PPIA forward 5′-GTTTGCAGACAAGGTCCCA-3′; PPIA reverse 5′-ACCCGTATGCTTTAGGATG-3′.

### Lentiviral shRNA infection

The Ha-*ras*^val12^ (TRCN0000040092) and RbAp46 (TRCN0000038885)-specific shRNA lentiviruses were designed and produced by the National RNAi Core Facility at the Institute of Molecular Biology, Academia Sinica, Taipei, Taiwan. The cells (3 × 10^5^) were seeded in a 10 cm cell culture dish and incubated in DMEM medium with 10% FBS overnight in a 5% CO_2_ incubator. Before viral infection, the cells were replaced with 5 ml culture medium containing 8 μg/ml polybrene (Sigma, St. Louis, MO, USA), and then the virus was added to cells. After 24 hr, the medium was removed from cells, and 5 ml fresh normal medium with 2 μg/ml puromycin (Sigma, St. Louis, MO, USA) was added to select cells for 48 hr.

### Promoter activity assay

The cells were plated onto 24-well plates (5 × 10^4^ cells/well) and grown overnight. The mouse RECK-luciferase plasmid pGL3-RECK (0.2 μg) and the internal control plasmid pRL-TK (0.025 μg) were co-transfected into the cells using Lipofectamine 2000 reagent (Invitrogen, Life Technologies, Inc., Grand Island, NY, USA). Luciferase activity was determined using the Dual-light Luciferase Assay System (Promega, Madison, WI, USA) according to the procedure suggested by the manufacturer and was normalized for *Renilla* luciferase activity. Ha-*ras*- and RbAp46-specific siRNA duplexes (Ha-*ras* 5′-TGGCTGCACGCACTGTGGAAT-3′; RbAp46 5′-CAAUCAGCAGA AGAUGCAU-3′), designed to target human Ha-*ras* and RbAp46 were synthesized from Qiagen (Carlsbad, CA, USA). Specific siRNAs were transfected into the cells using Lipofectamine 2000 reagent. Luciferase activity was determined 48 hr after transfection.

### Co-Immunoprecipitation

After various treatments, the cells were harvested in lysis buffer and cellular protein extracts (200 μg) were incubated with anti-RbAp46, anti-HDAC1 or anti-Sp1 antibodies at 4°C for 16 hr. Immuno-complexes were collected by adding 20 μl of protein A agarose beads (Amersham, Piscataway, NJ, USA). Samples were electrophoresed on 10% SDS polyacrylamide gels and transferred to poly- vinylidene fluoride (PVDF) membranes (Millipore, Billerica, MA, USA). Membranes were then reacted individually with anti-HDAC1 monoclonal antibody, anti-RbAp46 monoclonal antibody and anti-Sp1 polyclonal antibody.

### DNA affinity precipitation assay (DAPA)

DAPA was performed using streptavidin-coated beads to bind a biotinylated DNA probe, which was used to interact with nuclear extract proteins. The sequence of the DNA probe was 5′- GCGCCGGGGGCGGGGCCTGGTGCC-3′corresponding to the Sp1 site, originally designated as Sp1(B) in the mouse RECK promoter [12]. Nuclear extract proteins (200 μg) were incubated with 6 μg of biotinylated DNA probe and 45 μl of 4% streptavidin-coated beads at room temperature for 1 hr with constant shaking. After centrifugation, the beads were collected and washed three times with cold phosphate-buffered saline. Proteins bound to the beads were eluted with SDS-PAGE sample buffer and the binding proteins were resolved by 10% SDS-PAGE. Immunoblotting was performed as described above to examine the proteins bound to the DNA probe.

### Chromatin immunoprecipitation (ChIP) assay

The cells (2×10^6^ cells/10 cm plate) were treated with IPTG (5 mM, Invitrogen, Boston, MA, USA) for 24 hr, and ChIP assay was conducted as previously described [25]. Briefly, cells were crosslinked at 37°C for 5 min using 1% formaldehyde. After sonication, the resulting soluble chromatin was diluted 1:10 with ChIP dilution buffer and immunoprecipitated by anti-Sp1 antibody (Millipore, Billerica, MA, USA), anti-RbAp46 antibody (Abcam, Cambridge, MA, USA) or control IgG. The chromatin-antibody complexes were incubated with salmon sperm DNA/Protein A Agarose-50% (Millipore Corp., Billerica, MA, USA) overnight at 4°C with rotation. The DNA was eluted from the beads using ChIP elution buffer and purified by spin column. The primers used for detection of RECK promoter were as follows: forward: 5′-CAGCTGGCCCATAACAAAGA- 3′ and reverse: 5′-CGGCCAGCA GAAGTA GCA- 3′.

### Transwell^TM^ invasion assay

Cell invasion assay was performed in a 24-well Transwell™ (Costar, Cambridge, MA, USA). The upper chamber surface of the filter was coated with Matrigel (R&D systems, Minneapolis, MN, USA) before the experiment. The cells were prepared (3 × 10^5^/100 μl) with serum-free DMEM and loaded into the upper chamber. DMEM medium containing 10% FBS was added to the bottom chamber as the chemoattractant. After 18 hr incubation, wet cotton was used to remove the non-invaded cells from the wells. The cells were fixed with 1% formaldehyde for 15 min at room temperature, stained with 0.1% crystal violet for 15 min and quantified by counting the total number of cells in four independent areas under the light-field microscope.

### MMP-9 activity assay

The T24 cells with different treatments (5 × 10^5^/well) were seeded onto 12-well plates and filled with 500 μl medium for 48 hr. The supernatants were collected after centrifugation at 2500 rpm (4°C) for 10 min. MMP-9 activity was assessed by SensoLyte® 520 MMP-9 assay kit (ANASPEC, San Jose, CA, USA) according to the manufacturer’s instructions. The MMP-9 activity was monitored at excitation/emission = 490 nm/520 nm by fluorescence spectrometer (Thermo Scientific, Waltham, MA, USA).

### Experimental metastasis assay

Eight-week-old male BALB/c nude mice were obtained from the Laboratory Animal Center, National Cheng Kung University, Tainan, Taiwan, where they were maintained in a pathogen-free environment throughout the study. All animal experiment protocols were approved by the Institutional Animal Care and Use Committee (IACUC), National Cheng Kung University. The 7–4 cells, 7–4 cells treated with IPTG, or 7–4 cells carrying RbAp46 shRNA and treated with IPTG were injected through the tail vein into the nude mice (3 × 10^5^ cells/mice). There were three groups, each containing five mice. Two groups were feed with IPTG (12.5 mM) in their drinking water during the experimental period. After three weeks, the mice were sacrificed to evaluate the lung nodules, weight and protein expression.

### Cancer patient specimens

Four pairs of human primary bladder cancer and matched adjacent normal tissues were collected from Chiayi Christian Hospital, Chiayi City, Taiwan. All procedures were approved by the Institutional Review Board of Chiayi Christian Hospital (The approved No. IRB099058), with written informed consent from all donors. In addition, the specimens were coded and deleted of all of personal information.

### Statistical analysis

All data were expressed as means ± S.D. Student’s *t* test was used to analyze the significance. Spearman correlation was performed using GraphPad Prism 6 to show correlations among Ras, RbAp46 and RECK.

## Results

### Ras up-regulates RbAp46 expression in human cancer and mouse NIH 3T3 derivative cells

**Figure 1:**
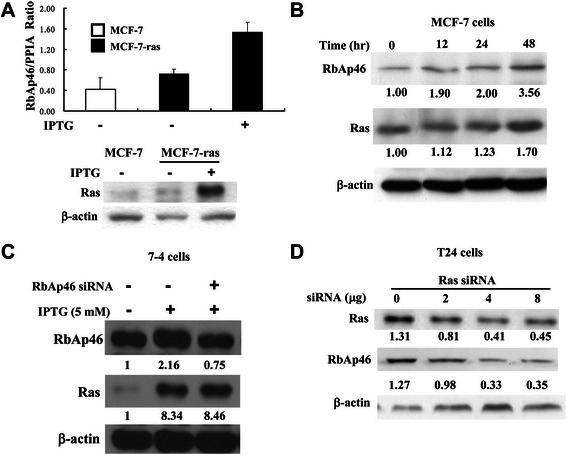
**Ras up-regulates RbAp46 expression in various human cancer cell lines and in mouse fibroblast 7–4 cells. (A)** MCF-7-*ras* human breast carcinoma cells containing an inducible Ha-*ras*^*val12*^ oncogene were treated with IPTG for 48 hr to induce Ras expression. Cells were harvested and the RNA was isolated for real-time RT-PCR analysis of RbAP46. Peptidylprolyl isomerase A (PPIA) was used for normalization of RbAp46 mRNA levels (upper panel). Ras expression induced by IPTG was confirmed by Western blotting using anti-Ras antibody (lower panel). β-actin was used as the internal control. **(B)** MCF-7-*ras* cells were treated with IPTG to induce Ras expression for various durations. Cell lysates were harvested and the expression levels of Ras and RbAp46 protein were evaluated by Western blotting using anti-Ras and anti-RbAp46 antibodies. The band intensity was quantified by a densitometer. **(C)** The 7–4 cells were treated with IPTG and transfected with different dosages of *ras* siRNA for 48 hr. The protein expression levels of Ras and RbAp46 were determined using the specific antibodies by Western blotting. c-Met siRNA was used as the negative control. **(D)** T24 cells were transfected with *ras-*specific siRNA at different dosages. After 48 hr, the protein expression levels of Ras and RbAp46 were determined.

We initially identified RbAp46 gene as a Ha-*ras*^val12^ up-regulated gene (2.7 fold increase) by suppression subtractive hybridization PCR screening in the uroepithelial cells T1R1 harboring the inducible Ha-*ras*^val12^ gene in the presence of the inducer IPTG (Additional file 2: Table S1). T1 also named BFTC905 was derived from a grade III, stage D1 transitional cell carcinoma (TCC) of urinary bladder [26]. We further showed that Ha-*ras*^val12^ expression was greatly induced compared to the un-induced control cells and the parent cells in the presence of IPTG. Accordingly, RbAp46 mRNA expression correlated with Ha-*ras*^val12^ expression was increased (Additional file 2: Figure S1A and S1B). We further demonstrated that Ras expression correlates with RbAp46 expression in the breast cancer cell MCF-7-*ras,* which harbors the same inducible Ha- *ras*^val12^ oncogene [21]. The increase of RbAp46 RNA and protein expression correlates with Ras protein induction in a time-dependent manner in the presence of IPTG in MCF-7-*ras* cells (Figure 1A and 1B). These results indicate that regulation occurs at the mRNA level. Mouse NIH3T3 derivative 7–4 cells, which are similar to MCF-7-*ras* cells also contain the same inducible Ha-*ras*^val12^ gene [22], were transected with *ras* siRNA for 48 hr. Under Ras-overexpressed conditions, the expression level of Ras protein was gradually decreased by *ras* siRNA in a dose-dependent manner. *c-Met* siRNA was used as a negative control. As with decreased Ras expression, the level of RbAp46 protein was also gradually decreased (Figure 1C). Similarly, in the bladder cancer T24 cells, Ras protein expression was decreased after transfection with *ras* siRNA at various dosages. RbAp46 protein expression was decreased in a Ras-dependent fashion, which was consistent with the aforementioned result (Figure 1D). The above data suggest that Ha-*ras*^val12^ up-regulation of RbAp46 is a general event. In summary, RbAp46 is a Ha-*ras*^val12^ up-regulated gene in various cells and the regulation occurs at the mRNA level.

### RbAp46 participates in the inhibitory action of Ras on RECK expression in T24 and 7–4 cells

**Figure 2:**
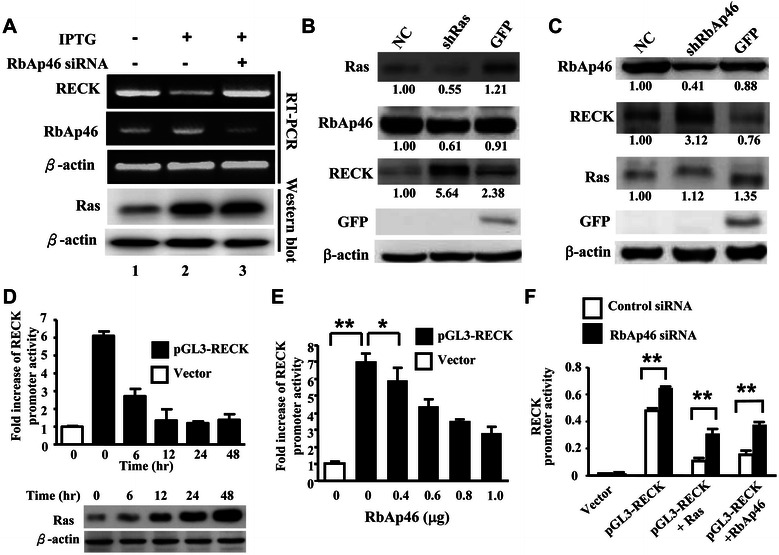
**RbAp46 participates in the inhibitory action of Ras on RECK expression in T24 and 7–4 cells. (A)** The 7–4 cells, transfected with or without RbAp46-specific siRNA (55.6 nM), were treated with or without IPTG for 48 hr. RT-PCR was performed using RECK- and RbAp46-specific primer sets to measure RECK and RbAp46 mRNA levels. β-actin was used as the internal control. Ras expression was confirmed by Western blotting. **(B)** T24 cells were infected with *Ras* shRNA lentivirus for 24 hr and selected by puromycin. A scrambled shRNA expressing GFP was used as the negative control. The protein expression of Ras, RECK, RbAp46 and GFP was determined by Western blotting. NC: negative control. **(C)** T24 cells were infected with RbAp46 shRNA lentivirus as described above. Expression of various proteins was detected by Western blotting using specific antibodies as indicated. NC: negative control. **(D)** The 7–4 cells that were seeded onto 24-well plates for 24 hr were transfected with RECK promoter-luciferase reporter plasmid (pGL3-RECK) followed by IPTG treatment to induce Ras expression for the times indicated. The cell lysates were harvested and luciferase activities were measured using the luciferase assay system (upper panel). Ras protein levels in the 7–4 cells were analyzed by Western blotting (lower panel). **(E)** The 7–4 cells were co-transfected with 0.2 μg of pGL3-RECK or Vector (pGL3) and pcDNA-RbAp46 plasmids at various amounts (0.4-1.0 μg). Luciferase activity was measured 48 hr after transfection. * indicates statistical significance at p < 0.05 and ** indicates p < 0.001 by Student’s *t* test analysis. **(F)** The 7–4 cells were co-transfected with 0.2 μg of either pGL3 vector, pGL3-RECK, pGL3-RECK + Ras or pGL3-RECK + RbAp46 cDNA, and RbAp46-specific siRNA (55.6 nM) or a control siRNA (55.6 nM). Luciferase activity was measured 48 hr after transfection.

Sasahara *et al.* and Chang *et al.* reported that oncogenic Ras decreases RECK expression through ERK pathway [12,13]. Moreover, RbAp46 as a core component of HDAC complex participates in the transcriptional repression [27,28]. Therefore, we hypothesize that RbAp46 may participate in Ras-induced suppression of RECK expression. Our data showed that IPTG induced Ras expression increased RbAp46 together with suppression of RECK mRNA expression (Figure 2A, lane 2) compared to that in cells without IPTG induction in 7–4 cells by RT-PCR (Figure 2A, lane 1). This inverse correlation was further confirmed by RbAp46 siRNA under Ras overexpression conditions (Figure 2A, lane 3). Using bladder cancer T24 cells, our data consistently showed that RbAp46 protein expression was decreased and RECK protein expression was increased when Ras was suppressed by Ras shRNA lentivirus (Figure 2B). To confirm whether RbAp46 is indeed involved in suppression of RECK expression, T24 cells were infected with RbAp46 shRNA. Our data showed that RECK expression was increased while RbAp46 expression was suppressed. As expected, Ras protein expression was not affected. The results indicate that RbAp46 negatively regulates RECK and Ras is an upstream regulator (Figure 2C). Taken together, the above data show that inhibition of either Ras or RbAp46 increases both RECK mRNA and protein expression, indicating that Ras negatively regulates RECK through RbAp46 at the transcriptional level.

To determine how Ras and RbAp46 regulate RECK expression, mouse RECK promoter reporter plasmid pGL3-RECK was transfected into 7–4 cells. The results showed that the decrease of RECK promoter activity inversely correlates with the gradual increase of Ras protein in a time-dependent manner (Figure 2D, lower panel vs. upper bar diagram). We further showed that the inhibition of RECK promoter activity was increased in a dose-dependent fashion in 7–4 cells by transfection of RbAp46 plasmid DNA (pcDNARbAp46) (Figure 2E). To further clarify the inhibitory effect of RbAp46 on RECK activity, RbAp46 siRNA was used. Consistently, RbAp46 siRNA significantly counteracted suppression of RECK promoter activity induced by endogenous RbAp46 (pGLC3-RECK only), Ras (pGLC3-RECK + Ras), and exogenous RbAp46 (pGLC3-RECK + RbAp46) (Figure 2F). The aforementioned findings demonstrate that Ras is an upstream regulator of RbAp46 which negatively regulates RECK expression at the transcriptional level.

### Ras overexpression increases association of RbAp46 with HDAC1 on SP1 site of RECK promoter in 7–4 cells

**Figure 3:**
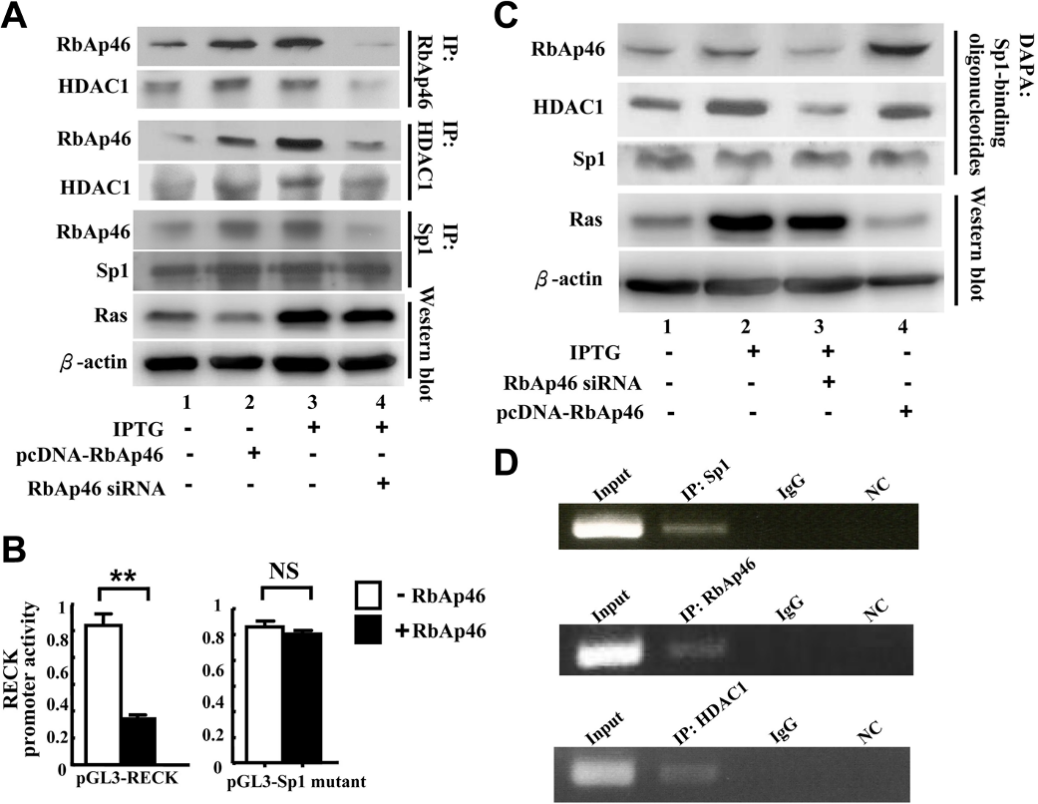
**Ras overexpression increases association of RbAp46 with HDAC1 at the SP1 site of RECK promoter in 7–4 cells. (A)** The 7–4 cells were transiently transfected with plasmid DNA pcDNA-RbAp46 (0.2 μg) or RbAp46-specific siRNA (55.6 nM) in the presence of IPTG for 48 hr. After treatment, co-immunoprecipitation was conducted using anti-RbAp46, anti-HDAC1 antibodies, or anti-Sp1 antibody. **(B)** The cells were co-transfected with 0.2 μg of plasmid DNA of pGL3-RECK and pGL3-Sp1 mutant in the presence or absence of pcDNA-RbAp46 plasmid. RECK promoter activity was measured at 48 hr post-transfection. **: statistical significance at p < 0.001 by Student’s *t* test analysis. **(C)** The 7–4 cells were transfected with pcDNA-RbAp46 (0.2 μg) or RbAp46-specific siRNA (55.6 nM) and incubated with IPTG for 48 hr. DNA affinity precipitation assay was performed using streptavidin-coated beads to bind the biotinylated DNA probe, which was used to interact with nuclear extract proteins. **(D)** The 7–4 cells were incubated with IPTG for 24 hr. Chromatin was extracted, and then immunoprecipitated by Sp1, RbAp46, or HDAC1 antibody. After elution, DNA samples were analyzed using PCR for Sp1 binding sequence in the RECK promoter region. NC: negative control.

It is known that RbAp46 is a component of HDAC-complex and Ras activation increases the binding of HDAC1 and Sp1 to the RECK promoter at the Sp1 site [13,15]. We showed above that Ras-induced suppression of RECK expression is through up-regulation of RbAp46. We therefore hypothesize that RbAp46 forms a complex with HDAC1 and then binds with Sp1 to regulate RECK expression via the Sp1 site of the promoter. We thus determined whether RbAp46 formed a complex with HDAC1 in 7–4 cells by treatment with IPTG to induce Ras expression. The results showed that the formation of RbAp46-HDAC1 complex was increased compared to that in the cells without IPTG treatment as demonstrated by co-immunoprecipitation assay (Figure 3A, lane 3 vs. lane 1). In addition, exogenous expression of RbAp46 (pcDNA-RbAp46) also increased RbAp46-HDAC1 complex formation (Figure 3A, lane 2). The reverse effect of complex formation was demonstrated in the cells treated with RbAp46 siRNA to silence RbAp46 (Figure 3A, lane 4). In contrast, Sp1 was steadily co-immunoprecipitated with RbAp46 and HDAC1, suggesting that Sp1 is not regulated by Ras as well as RbAp46 and may function as a docking protein for HDAC1-RbAp46 complex formation. Furthermore, to confirm that RbAp46 suppression of RECK promoter activity occurs through the Sp1-binding site of the RECK promoter, the Sp1-binding site mutant of RECK promoter reporter plasmid (pGL3-Sp1 mutant) was used. Our data showed that no suppression of the RECK promoter was detected with the mutated Sp1-binding site as compared with the control (pGL3-RECK) in the presence of RbAp46 (Figure 3B). This result indicates that RbAp46 binds with HDAC1 and Sp1 to regulate RECK promoter at the Sp1 site.

Binding of RbAp46 complex to the RECK promoter was further confirmed by DNA affinity precipitation assay (DAPA) using a DNA probe of RECK promoter containing Sp1 binding site. The amount of RbAp46 and HDAC1 on the complex binding to the Sp1 site was increased when the RbAp46 expression level was increased either by increased Ras expression (Figure 3C, lane 2) or by increased RbAp46 expression (Figure 3C, lane 4). This phenomenon was abolished when RbAp46 siRNA was introduced into the cells in the presence of IPTG (Figure 3C, lane 3). Control cells without IPTG treatment showed basal levels of RbAp46, HDAC1 and Sp1 binding (Figure 3C, lane 1). To confirm that the RbAp46 complex indeed binds to the RECK promoter at the Sp1 binding site *in vivo*, a ChIP assay was conducted. The chromatin of 7–4 cells was extracted after IPTG treatment for 24 hr. The lysate was immunoprecipitated by Sp1, RbAp46, and HDAC1-specific antibodies. After elution, the precipitated DNA samples were screened by PCR for Sp1 binding sequence in RECK promoter region. Our data showed that the Sp1 sequence in the RECK promoter was amplified in RbAp46, HDAC1, and Sp1 precipitated DNA samples under Ras-overexpression conditions (Figure 3D). Taken together, the above results indicate that RbAp46 forms a complex with HDAC1 and Sp1, which binds to the Sp1 binding site in the RECK promoter to suppress RECK expression in the cell.

### Suppression of RbAp46 decreases cell invasion and negatively regulates MMP-9 activity

**Figure 4:**
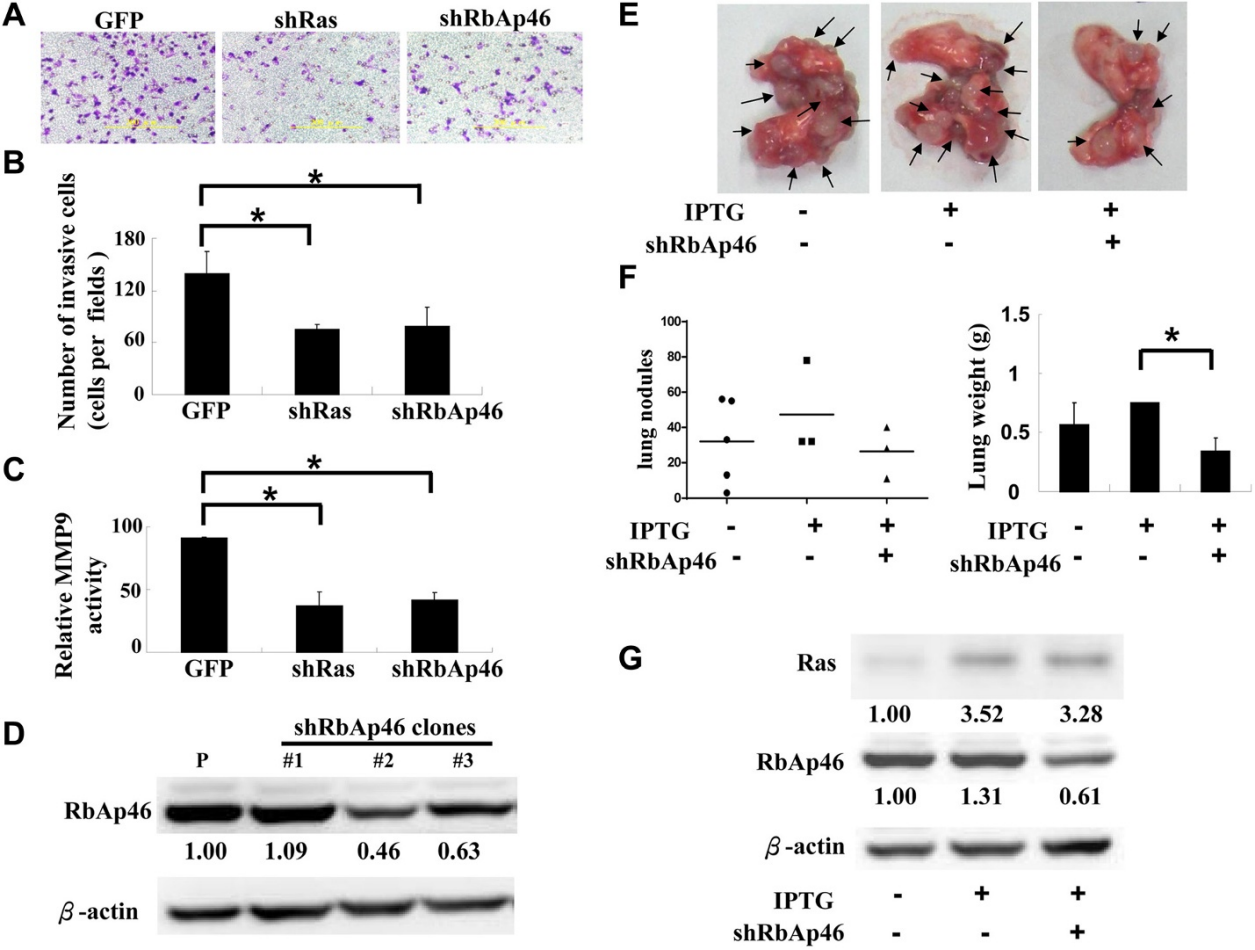
**Silencing Ras or RbAp46 expression decreases invasive activity and lung metastasis of the bladder cancer T24 cells in nude mice. (A)** T24 cells treated with shRNA of Ras, RbAp46 or GFP lentivirus for 24 hr were seeded into matrigel-coated Transwell™ wells. Medium containing 10% FBS was used as chemoattractant. The cells were incubated for 18 hr and the invasive cells were stained, photographed and counted. **(B)** The quantification of invading cells was shown as mean ± S.D. of 9 samples from 3 independent experiments. Student’s *t* test was used, *p < 0.05. **(C)** T24 cells were treated with shRNA of Ras, RbAp46 or GFP lentivirus for 24 hr. The supernatants were centrifuged and collected. MMP-9 activity was measured using the SensoLyte® 520 MMP-9 assay kit. Data represent the results as mean ± S.D. of three independent experiments. Student’s *t* test was used, *p < 0.05. **(D)** The 7–4 cells were infected with different RbAp46 shRNA lentiviral clones. The clones with significant silencing of RbAp46 were selected. The protein expression was evaluated by Western blotting using specific antibodies. P: parental cells. **(E)** The 8-week-old male BALB/c nude mice were injected intravenously with 7-4-shRbAp46 (#2) cells (3×10^5^) through the tail vein. Mice were sacrificed three weeks later and the lungs were collected, weighted and photographed. Arrows indicate lung nodules. **(F)** The lung weight and the number of lung nodules from E were quantified. **(G)** The lung proteins of those mice were extracted and expression levels of Ras and RbAp46 of five mice in each group were detected by Western blotting. Each lane represented a group of mice. *indicates statistical significance at p < 0.05 by Student’s *t* test analysis.

Chang *et al.* reported that RECK expression inhibits cell invasion and metastasis [29]. Since RbAp46 negatively regulates RECK expression, the effect of RbAp46 on cell invasion was investigated. Bladder cancer T24 cells were infected with Ras or RbAp46 shRNA lentivirus. The GFP-containing lentivirus was used as a positive control for lentivirus infection efficiency and a negative control for the experiment. Inhibition of Ras or RbAp46 by shRNA lentiviruses significantly decreased T24 cell invasion compared with the cells infected with GFP lentivirus (Figure 4A and 4B). MMP-9 is one of the matrix metalloproteinases (MMPs) responsible for cell invasion [7]. Chang *et al.* reported that inhibition of invasion by RECK is achieved by decreasing MMP-9 activity [29]. Therefore, we investigated whether MMP-9 activity is regulated by Ras or RbAp46 in T24 cells. After inhibition of Ras or RbAp46 by shRNA, cell culture supernatants were collected to evaluate MMP-9 activity using the MMP-9 assay kit SensoLyte® 520. The results showed that MMP-9 activity was greatly decreased (>50%) in the infected T24 cells by either Ras or RbAp46 shRNA compared with that in the cells infected with GFP alone, indicating that RECK affects MMP-9 activity through Ras or RbAp46 (Figure 4C). Our results suggest that Ras up-regulates RbAp46 to suppress RECK expression, which leads to increased MMP-9 activity and cell invasion.

### Suppression of RbAp46 decreases metastasis of 7–4 cells in the xenograft BALB/c nude mice model

The above *in vitro* data showed that suppression of RbAp46 decreases cell invasion and inhibits MMP-9 activity. The role of RbAp46 *in vivo* in Ras-induced metastasis was then investigated. The 7–4 cells were infected with RbAp46 lentiviral shRNA and stable clones were selected. RbAp46 shRNA lentiviral clone #2 showed the best suppression of RbAp46 (Figure 4D). The cells with or without IPTG and/or RbAp46 shRNA were injected intravenously (i.v.) into male BALB/c nude mice. The mice were divided into three groups (n = 5). Two groups (7–4 and 7-4-shRbAp46 #2) were fed with IPTG water for three weeks. Tumor nodules were evaluated on day 21 after injection. When Ras expression was induced by IPTG, suppression of RbAp46 expression by shRNA showed fewer nodules in the lung compared with the number of nodules found in the other groups (Figure 4E). Similarly, inhibition of RbAp46 reduced lung weight compared with that in the other groups (Figure 4F). The lungs of five mice from each group were combined and protein extracts prepared to determine the expression levels of Ras and RbAp46 by Western blotting (Figure 4G). The results showed that the expression level of Ras was induced by IPTG (Figure 4G, lane 2) and RbAp46 was inhibited by RbAp46 shRNA (Figure 4G, lane 3) in mice compared with those in the control group (Figure 4G, lane 1). In summary, our data demonstrate that inhibition of RbAp46 by shRNA in 7–4 cells decreased the number of metastastic nodules induced by Ras in the nude mice model. Taken together, we reveal that Ras-related metastasis occurs through up-regulation of RbAp46 in the xenograft mouse model.

### Interrelationships among Ras, RbAp46 and RECK in clinical bladder cancer tissues

**Figure 5:**
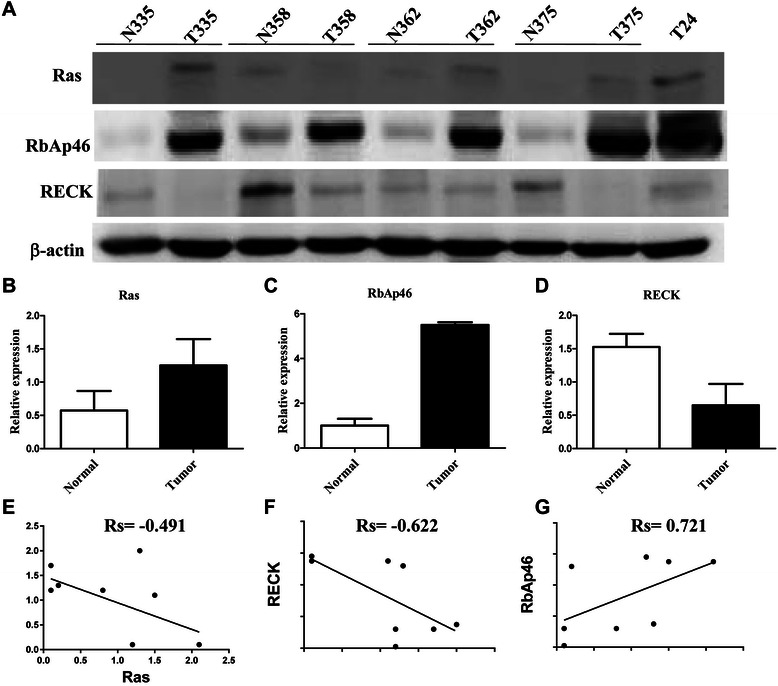
**Interrelationships among Ras, RbAp46 and RECK in clinical bladder cancer tissues. (A)** The proteins extracted from the tissue of four clinical bladder cancer patients were evaluated by Western blotting to detect expression levels of Ras, RECK and RbAp46 using specific antibodies as indicated. β-actin was used as the internal control. N: normal tissue of bladder, and T: bladder tumor in patients. T24 cells were used as a positive control for the expression of mutant Ras and RECK. The intensity of bands for Ras **(B)**, RECK **(C)** and RbAp46 **(D)** was quantified by a densitometer. Quantified intensity of bands was plotting using a Spearman correlation **(E-G)**.

To further clarify the relationships among mutant Ras (active Ras), RbAp46 and RECK in clinical bladder cancer, the expression levels of these three proteins in bladder cancer specimens were evaluated by Western blotting. High expression of Ras protein and RbAp46 protein and low expression of RECK protein were detected in bladder cancer specimens (Figure 5 A-D). Our data show that high RbAp46 expression was inversely correlated with low RECK expression in the tumor tissues compared to the adjacent non-tumor parts in all of 4 specimens analyzed (100%) (Figure 5A). Moreover, high expression of Ras protein together with high RbAp46 expression were detected in 75% of tested specimens (3/4) (Figure 5A). In summary, we have demonstrated a correlation among Ras-RbAp46-RECK in bladder cancer specimens (Figure 5E-5G), indicating the importance of Ras, RbAp46 and RECK in bladder tumorigenesis.

## Discussion

Using a TCC bladder cancer cell line harboring an inducible Ha-*ras*^val12^ gene [26], we identified and confirmed that RbAp46 is a Ras-induced gene by SSH PCR and Northern blotting, respectively (Additional file 2: Figure S1A and Table S1). This relationship between Ras and RbAp46 was further confirmed in the NIH3T3 derivative 7–4 cells, which also contain the inducible Ha-*ras*^val12^ gene [30-32] (Additional file 2: Figure S1B). In this study, we revealed that Ras-induced RbAp46 forms a complex with HDAC1 and Sp1, which further binds to the Sp1 site of the RECK promoter and suppresses its expression. The relationship among Ras, RbAp46 and RECK expression was also detected in 75% (3/4) of the bladder cancer specimens analyzed. This is the first report to show that RbAp46 as a Ras up-regulated gene plays a pivotal role in Ras-related lung metastasis through binding of the RbAp46 with HDAC1 and Sp1 to suppress RECK expression and activate MMP9. NIH3T3 fibroblasts were used in this study, because of their ease to be transfected and transformed by oncogenes.

Ahringer *et al.* reported that RbAp46 is a core component of the HDAC-SIN3-NuRD complex, which participates in gene regulation through interaction with specific co-repressors [15]. Recruitment of HDACs to promoter regions is a general mechanism of transcriptional repression of the target genes in cells. For example, the recruitment of HDACs to specific promoters could be mediated through direct interactions with regulatory proteins such as retinoblastoma (Rb) and metastatic-associated protein 1 (MTA1) [33-36]. Chang *et al*., reported that Ras overexpression increases the binding of HDAC1 to the Sp1 site and suppresses RECK (metastasis inhibitor) expression [13]. Here, we further revealed that RbAp46 utilizes Sp1 as a docking protein to recruit HDAC1 and RbAp46 and suppresses RECK expression via the Sp1 binding site. This result indicates that the increased binding of the RbAp46 with HDAC1 and Sp1 to the Sp1 site is dependent on the protein level of RbAp46, which is possibly responsible for the suppression of RECK expression. However, whether other processes of epigenetic regulation such as acetylation or methylation are involved along with complex formation requires further investigation. Jaejoon *et al.,* reported that Sp1 and Sp3 form a complex with HDAC2 to repress human telomerase reverse transcriptase (hTERT) expression [37]. Therefore, the possibility of the recruitment of HDAC2 by RbAp46 can not be excluded. Moreover, RbAp46 is an Rb-binding protein, so it is possible that RbAp46 could interact with Rb protein and recruit HDAC1 to the promoter.

RbAp46 overexpression induces epithelial-mesenchymal transition (EMT) and increases the migration and invasion of MCF10AT3B mammary epithelial cells [19]. Wang *et al*., showed that elevated serum RbAp46 levels are highly correlated with non-small cell lung cancer (NSCLC) distant metastasis and knockdown of RbAp46 inhibits the migration ability of lung cancer cells [20]. Those reports indicate that RbAp46 participates in EMT, cell migration, and invasion as well as tumor metastasis. However, there is no *in vivo* evidence to show the effect of RbAp46 on cancer metastasis. Our experimental metastatic mouse model provides the first evidence that RbAp46 participates in Ha-*ras*^val12^-induced lung metastasis. Our finding that RbAp46 suppresses RECK expression and decreases Ha-*ras*^val12^-induced lung metastasis is consistent with the results of other reports which showed that RECK protein reduces metastasis and that down-regulation of RECK is correlated with lymph node metastasis of lung cancer cells [29,38]. When RECK is down-regulated, the activity of MMP-9, a key enzyme involved in tumor invasion and metastasis, is increased [5]. We also demonstrated that the RbAp46 expression level positively correlates with MMP-9 activity in T24 cells (Figure 4C). Our results provide a mechanistic link for RbAp46 regulated cell invasion and metastasis. Collectively, RbAp46 enhanced cell invasion and metastasis are possibly caused by increased activity of MMP-9 resulting from RECK suppression.

When oncogenic Ras is highly expressed, many down-stream signaling pathways are activated such as Raf-1, PI3K and Ral-GDS [2]. Previous studies showed that oncogenic Ras and HER-2/Neu down-regulate RECK expression through MEK/ERK signaling [12,13]. We also revealed that Ha-*ras*^val12^ transcriptionally up-regulate RbAp46 (Additional file 2: Figure S2A) through the Raf-mediated MEK/ERK signaling pathway (Additional file 2: Figure S2B). A previous study showed that Raf-MEK-ERK1/2 pathway induced by Ha-*ras*^val12^ is necessary for experimental metastatic ability of NIH3T3 cells [39]. Welch *et al*., further showed that MEK1 activation leads to the production of MMP-2 and MMP-9 which contribute to Ras-mediated metastatic potential [40]. Therefore, Raf/MEK/ERK signaling may play a critical role in Ras-mediated RECK suppression and metastasis through up-regulation of RbAp46. However, the detailed mechanism of how RbAp46 is regulated remains to be determined. In T24 cells, RECK protein increased dramatically after Ras knockdown, however, there were no obvious changes in RbAp46 (Figure 2B). This finding suggests a parallel mechanism of Ras-induced RECK regulation. Chang *et al*. reported that Ras-induced down-regulation of RECK is mediated via a DNMT3b/promoter methylation mechanism [10]. Loayza-Puch *et al.* reported that Ras-signaling pathways could downregulate RECK protein through microRNAs [11]. Whether DNMT3b and microRNA regulate Ras-induced RECK downregulation independently of RbAp46 in T24 cells is worthy of investigation. In one bladder cancer specimen (case #358), Ras showed little difference between normal and tumor, and yet RbAp46 was strongly upregulated in the tumor. It is possible that endogenous Ras is regulated at the level of GDP-GTP exchange rather than altered protein expression.

Interestingly, RbAp46 is also a potent cell growth inhibitor that can suppress the transformed phenotype of tumor cells [17,18,41]. It is counterintuitive that a tumor suppressor promotes malignant behaviors. RbAp46 is an Rb-binding protein and has been proved to be a metastasis-associated protein 1 (MTA1) complex [42,43]. Expression of MTA1 correlates well with the metastasis potential of human cancers [44,45]. We observed that RbAp46 can directly interact with HDAC1. Therefore, it is possible that RbAp46 could promote tumor metastasis by regulating transcriptional suppression on RECK gene through histone deacetylation activity. However, the underlying mechanism needs to be further studied. It has been shown that RbAp46 expression inhibits the transformation of tumor cells by interfering with the normal cell cycle and/or enhancing apoptotic cell death [17]. We also found that overexpression of exogenous RbAp46 suppressed cell proliferation and decreased Ras-related tumor formation (unpublished data), which is consistent with previous findings [17,18,41]. RbAp48, another member of a subfamily of nuclear WD-repeat proteins, could interact with HDAC1 and then be recruited by Rb/E2F1 to the promoters of E2F-regulated genes during the G1 phase of the cell cycle [46]. It is possible that RbAp46 may utilize similar mechanisms to suppress cell proliferation and tumor formation.

In this study, we reveal that Ras up-regulates RbAp46 expression in human breast and bladder cancer cells as well as in mouse fibroblast 7–4 cells, and the binding of RbAp46 with HDAC and Sp1 represses RECK promoter activation via the Sp1 site. Taken together, RbAp46-induced down-regulation of RECK expression increases cell invasion and metastasis and is accompanied with promotion of MMP-9 activity. Finally, RbAp46 expression was found to be associated with mutant Ras expression and was inversely correlated with the expression of RECK in bladder cancer tissues. In conclusion, our findings provide a novel mechanism of Ras-related tumorigenesis in bladder cancer and suggest that RbAp46 could be a target in the treatment of Ras-related metastasis in bladder cancer.

## Conclusions

**Figure 6:**
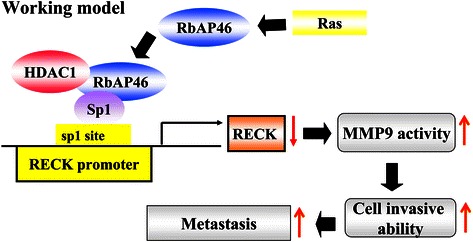
**A schematic hypothetical model shows that RbAp46 is a Ras up-regulated gene which participates in Ras-induced experimental lung metastasis through binding with HDAC1 and Sp1 to suppress RECK expression followed by MMP-9 activation and metastasis.**

In this study, we demonstrated that Ras-induced RbAp46 expression forms a complex with HDAC1 and Sp1, which further binds to the Sp1 site of the RECK promoter and suppresses RECK expression. The expression levels of Ras, RbAp46 and RECK were also determined in human bladder cancer specimens. Our working hypothesis is that RbAp46 is a Ras up-regulated gene which participates in Ras-induced experimental lung metastasis through binding with HDAC1 and Sp1 to suppress RECK expression followed by MMP-9 activation and metastasis (Figure 6). Our findings suggest a possible therapeutic strategy against Ras-related cancers via RbAp46 targeting.

## Additional files

Additional file 1: **Supplementary Materials and Methods.** Cloning of full-length RbAp46 promoter; Cancer Patient Specimens.

Additional file 2: Figure S1. RbAp46 is up-regulated by Ha-rasVal12 oncogene in T1R1 human bladder cancer cell line and mouse fibroblast 7-4 cells. After the cells were treated with IPTG (5 mM) for 48 hr, the expression levels of RbAp46 and Ras in bladder cancer cell T1R1 and the parental cell T1were evaluated by Northern blotting. T1R1 harbors the inducible Ha-rasval12 gene, and the parental T1 cells also named BFTC905 was derived from a grade III, stage D1 transitional cell carcinoma of urinary bladder (Tzeng et al.). β-actin was as used as the internal control. **Figure S2.** Ha-rasVal12 enhances RbAp46 promoter activity through MEK/ERK signaling pathway. (A) The pGL3-RbAp46-E6 and -R2 of RbAp46 reporter plasmids were co-transfected with pBSSK (1 μg) or pSGRas (1 μg) into HEK293 cells and the luciferase activities were determined after 48 hr. The pGL3-Basic was used as a negative vector control and pY2 containing the multiple Ets binding sites which could be activated by Ras was used as a positive control. (B) Inhibitors SB203580 (10 μM, for p38), PD98059 (20 μM, for MEK) and SP600125 (20 μM for JNK) were added into HEK293 culture medium 16 hr after transfection with pGL3-RbAp46-E6, -R2. Promoter activity was determined by luciferase activity assay 48 hr after transfection. **Table S1.** Ras up-regulated genes screened by suppression substractive hybridization PCR screening.
