# Supplementary material for: Ras induces experimental lung metastasis through up-regulation of RbAp46 to suppress RECK promoter activity
Source: BMC Cancer. 2015 Mar 25;15:172. doi: 10.1186/s12885-015-1155-7 (PMC4377201; doi:10.1186/s12885-015-1155-7)
Supplement: Supplementary file 1 — Supplementary Materials and Methods. Cloning of full-length RbAp46 promoter; Cancer Patient Specimens. [file 12885_2015_1155_MOESM1_ESM.doc]

**Supplementary Materials and Methods**

**Cloning of full-length RbAp46 promoter**

Genomic DNA extracted from transitional cell papilloma RT4 cells and HPV E7 immortalized uroepithelial cells were used to amplify RbAp46 promoter (-1427 to +83) using PCR. The conditions for PCR were 335 cycles of denaturation (94℃/1 min), annealing (55℃/1min), extension (72℃/1 min), and 1 cycle of final extension (72℃/10 min). The primers used were: RbAp46 forward 5’-CCTAGCTAGCACCCTTCCAACTCTCTTCCCTCTC-3’ and RbAp46 reverse 5’-CCCCAAGCTTGCGCTCTTCTCTCTCTCTCCAAAC-3’. The *Nhe*I and *Hind*III sites were used to clone the DNA fragment into the pGL3-basic vector (Promega, USA) to create pGL3-RbAp46-R2 and pGL3-RbAp46-E6.

**Cancer Patient Specimens**

Four pairs of human primary bladder cancer and matched adjacent noncancerous tissues were collected from Chiayi Christian Hospital, Chiayi, Taiwan. All procedures were approved by the Institutional Review Board at Chiayi Christian Hospital.
