# Supplementary material for: Ras induces experimental lung metastasis through up-regulation of RbAp46 to suppress RECK promoter activity
Source: BMC Cancer. 2015 Mar 25;15:172. doi: 10.1186/s12885-015-1155-7 (PMC4377201; doi:10.1186/s12885-015-1155-7)
Supplement: Supplementary file 2 — RbAp46 is up-regulated by Ha-rasVal12 oncogene in T1R1 human bladder cancer cell line and mouse fibroblast 7-4 cells. After the cells were treated with IPTG (5 mM) for 48 hr, the expression levels of RbAp46 and Ras in bladder cancer cell T1R1 and the parental cell T1were evaluated by Northern blotting. T1R1 harbors the inducible Ha-rasval12 gene, and the parental T1 cells also named BFTC905 was derived from a grade III, stage D1 transitional cell carcinoma of urinary bladder (Tzeng et al.). β-actin was as used as the internal control. Figure S2. Ha-rasVal12 enhances RbAp46 promoter activity through MEK/ERK signaling pathway. (A) The pGL3-RbAp46-E6 and -R2 of RbAp46 reporter plasmids were co-transfected with pBSSK (1 μg) or pSGRas (1 μg) into HEK293 cells and the luciferase activities were determined after 48 hr. The pGL3-Basic was used as a negative vector control and pY2 containing the multiple Ets binding sites which could be activated by Ras was used as a positive control. (B) Inhibitors SB203580 (10 μM, for p38), PD98059 (20 μM, for MEK) and SP600125 (20 μM for JNK) were added into HEK293 culture medium 16 hr after transfection with pGL3-RbAp46-E6, -R2. Promoter activity was determined by luciferase activity assay 48 hr after transfection. Table S1. Ras up-regulated genes screened by suppression substractive hybridization PCR screening. [file 12885_2015_1155_MOESM2_ESM.pdf]

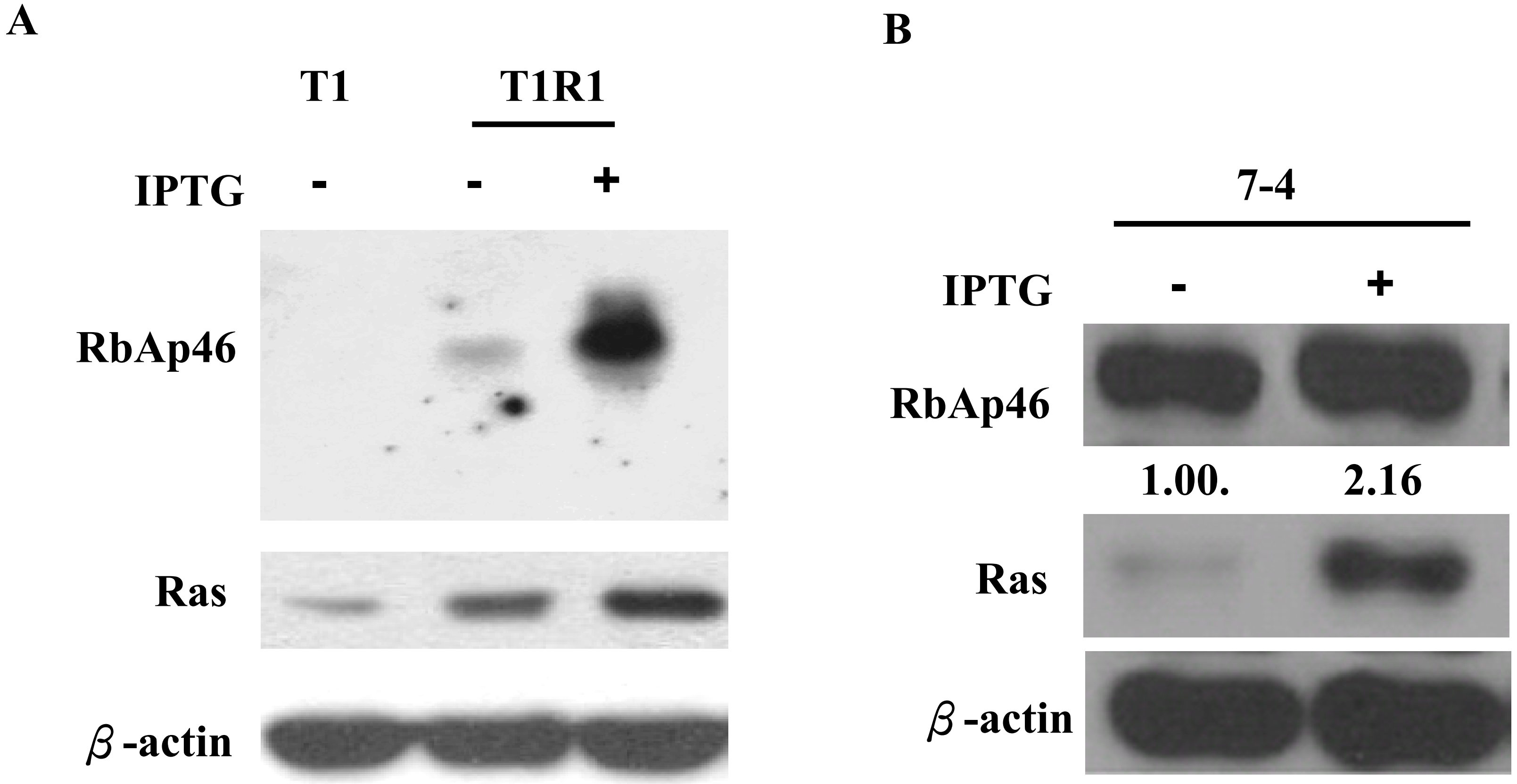

Supplementary Figure S1

A

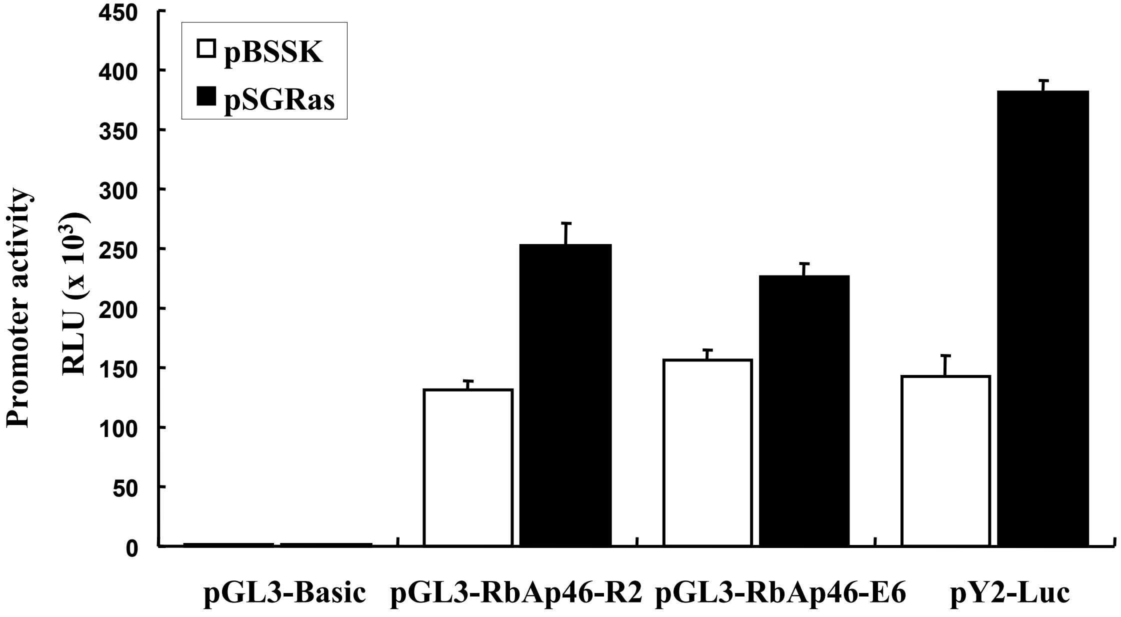

B

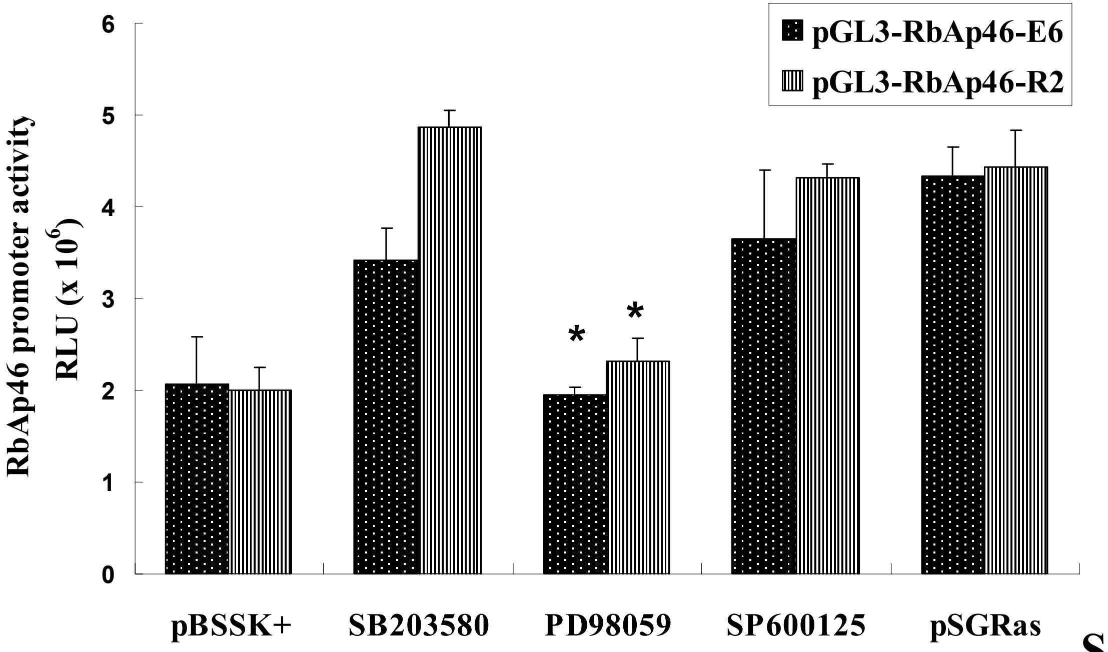

Supplementary Figure S2

Table S1. Ras up-regulated genes screened by suppression subtractive hybridization PCR screening

Name	Accession	UG_Link	Gene_Symbol	Function	Ratio of mean
RbAp46	NM_002893	Hs.406078	N/A	Negative regulation of transcription from Pol II promoter	2.7430104
Lutheran blood group	P50895	Hs.155048	Lu	N/A	2.622386
B-cell translocation gene 1, anti-proliferative	N70463	Hs.77054	BTG1	Cell cycle	2.5967418
Protein kinase C, nu	AA463213	Hs.143460	PRKCN	Kinase	2.5919929

Supplementary Table S1
